# Changing characteristics of the empathic communication network after empathy-enhancement program for medical students

**DOI:** 10.1038/s41598-018-33501-z

**Published:** 2018-10-10

**Authors:** Je-Yeon Yun, Kyoung Hee Kim, Geum Jae Joo, Bung Nyun Kim, Myoung-Sun Roh, Min-Sup Shin

**Affiliations:** 1 Yeongeon Student Support Centre, Seoul National University College of Medicine, Seoul, Republic of Korea; 2 Seoul National University Hospital, Seoul, Republic of Korea; 3 Department of Psychiatry and Behavioural Science, Seoul National University College of Medicine, Seoul, Republic of Korea; 4 Department of Medicine, Seoul National University College of Medicine, Seoul, Republic of Korea

## Abstract

The Empathy-Enhancement Program for Medical Students (EEPMS) comprises five consecutive weekly sessions and aims to improve medical students’ empathic ability, an essential component of humanistic medical professionalism. Using a graph theory approach for the Ising network (based on *l*_1_-regularized logistic regression) comprising emotional regulation, empathic understanding of others’ emotion, and emotional expressivity, this study aimed to identify the central components or hubs of empathic communication and the changed profile of integration among these hubs after the EEPMS. Forty medical students participated in the EEPMS and completed the Depression Anxiety Stress Scale-21, the Empathy Quotient-Short Form, the Jefferson Scale of Empathy, and the Emotional Expressiveness Scale at baseline and after the EEPMS. The Ising model-based network of empathic communication was retrieved separately at two time points. Agitation, self-efficacy for predicting others’ feelings, emotional concealment, active emotional expression, and emotional leakage ranked in the top 20% in terms of nodal strength and betweenness and closeness centralities, and they became hubs. After the EEPMS, the ‘intentional emotional expressivity’ component became less locally segregated (*P* = 0.014) and more directly integrated into those five hubs. This study shows how to quantitatively describe the qualitative item-level effects of the EEPMS. The key role of agitation in the network highlights the importance of stress management in preserving the capacity for empathic communication. The training effect of EEPMS, shown by the reduced local segregation and enhanced integration of ‘intentional emotional expressivity’ with hubs, suggests that the EEPMS could enable medical students to develop competency in emotional expression, which is an essential component of empathic communication.

## Introduction

The work of physicians requires an understanding of patients’ thoughts and emotional experiences, in addition to the ability to effectively communicate medical information^1–3^. Accordingly, empathy, which is a multi-component, socio-emotional relationship skill that is communicated through both verbal and nonverbal behaviours, is particularly important in the medical context. Behavioural expression of empathy from one party is perceived as warmth by a counterpart^4^. For example, through nonverbal empathic behaviours such as an open body posture (uncrossed arms), eye contact, and smiling, physicians convey warmth, empathy, and competency to their patients^5^. Empathy can also foster among physicians a more understanding approach to issues related to social prejudice or stereotypes (e.g., in relation to obese patients)^6^. Physicians’ empathy-based communication should foster patients’ trust in and satisfaction with their doctors^7,8^, increase treatment adherence^9^, and improve diagnostic accuracy^10^, leading to a more successful treatment response^11–13^. During medical school and/or residency training, physicians’ empathic capacity can be enhanced^14,15^, preserved^16^, or even eroded^17–19^. In a medical context, empathy is modulated by physician-related features such as their moral profile^20–22^, medical specialty orientation in medical school or residency training^23,24^, personality characteristics^25^, ethnicity^26–29^, gender and marital status^14^. In addition, physicians’ perceived distress and burnout are reflected in emotional exhaustion and depersonalization^30^, which might be affected by their workplace^31^, their degree of medical expertise^32^, or a lack of reward for personal achievements^33^ and affect their empathic behaviour.

A network-based approach could be a suitable and novel way to determine the essential drivers of physicians’ empathy and to perform finer-grained investigations of the effects of empathic communication training. A network of psychological characteristics consists of a collection of nodes (=variables of interest, such as individual questionnaire items) and edges (=dependency or associations between nodes)^34^. Relationships among psychological symptoms or psychopathologies, identified as present (=1) or absent (=0) in an individual, have been successfully estimated using the (*l*_1_-regularized logistic regression-based) Ising model network^34–37^. The capacity of each component to influence (or be influenced by) others in the Ising model-based network of empathic communication can be measured using regional network characteristics such as nodal strength (=sum of the absolute value of the edge weights connected to a specific node in the undirected-weighted network), betweenness centrality (=chances that a specific node of interest is located in the shortest path connecting two other nodes in a given network, reflecting the importance of a given node as a facilitator of information flow through the network), and closeness centrality (=average distance between a specific node and all other nodes in a given network)^34,38,39^. Higher-ranked components for these local network topology measures or influences in this network are also called hubs^34^. Moreover, previous studies have examined longitudinal changes in symptom-symptom interaction with a single group of patients using estimations of psychological networks combined with a graph theory approach^40,41^.

Recent meta-analyses have shown that empathy training can be successful^42,43^. However, few studies have used the network-based approach to examine training effects for the conceptual integration of multi-dimensional components that comprise empathic communication^42,43^. Therefore, using a graph theory approach for the Ising model-based empathic communication network, this study aimed to (1) find the most influential components (=hubs) of empathic communication in medical students and (2) examine the effect of the Empathy-Enhancement Program for Medical Students (EEPMS) on enhancing the inter-connectedness among the hubs of empathic communication. The EEPMS was constructed by the research team on the systemization of humanism education at the Seoul National University (SNU) College of Medicine^44^. The program aims to improve medical students’ empathic ability through five consecutive weekly sessions of extracurricular small-group activities involving, during which all of the elements required for efficient empathic communication in diverse everyday life situations and patient-doctor relationships are practiced through peer discussions, lectures, and role play, combined with real-time feedback (Table 1). Empathy is a multi-dimensional skill that works by way of interactions among moral (=physicians’ intrinsic motivation for empathic behaviour), affective (=sharing others’ emotions)^45^, cognitive (=understanding others’ emotions)^46^, social-contextual^47–49^, and behavioural (=expressing feedback to others) components^5,50–53^. All of these components were measured using the Empathy Quotient-Short Form (EQ-short; cognitive, emotional, and social subdomains of empathy)^54–56^, the Jefferson Scale of Empathy (JSE-S; patient care-related facets of empathy, such as perspective taking, standing in the patient’s shoes, and compassionate care)^57–59^, and the Emotional Expressivity Scale (EES; several forms of perspective or emotional expressiveness, both verbal and nonverbal)^60,61^ at baseline and after the completion of the five-week EEPMS. In addition, we assessed perceived distress and burnout using the Depression Anxiety Stress Scale-21 (DASS-21), which measures stress, depressive symptoms, and anxiety – factors that could deteriorate medical students’ empathic behaviour^30^. We first hypothesized that distress-related components could be ranked as hubs of the empathic communication network. Second, since the EEPMS is mainly based on practice, role play, and feedback, we hypothesized that behavioural components of the empathic communication network might show a significant reduction in the clustering coefficient (=a regional network measure of connectedness between a given node and its direct neighbours in local networks^41,62^) and a shorter path length (=number of steps required to be reached) with hubs of the network after the completion of EEPMS compared to baseline. Prior graph theory-based network approaches that measured and compared a single group of participants at baseline and at follow up successfully showed progressing patterns of the association between psychopathology at the acute and chronic phases after exposure to trauma^40^ as well as the altered properties of brain structure or functional connectivity networks before versus after a therapeutic intervention for brain tumours^63^ and mood disorders^64^, among others.

**Table 1:** Key components of the Empathy-Enhancement Program for Medical Students.

	Themes and detailed contents of discussion and role play
1^st^ session	■ Survey before program
	■ Mindfulness: how to monitor and recognize one’s condition
	■ Emotion recognition: how to distinguish feelings from thoughts
	■ Emotional expression using ‘I’ messages
2^nd^ session	■ Recognizing others’ emotions: how to decode nonverbal cues of emotion
	■ How to listen to others’ emotions: facilitative listening
	■ Cognitive chain of emotional response: situation, autonomic responses/thoughts, actions (thought, emotion, behaviour)
3^rd^ session	■ How to find cognitive biases and maladaptive emotional responses
	■ How to correct cognitive biases and maladaptive emotional responses
4^th^ session	■ The meaning and purpose of empathic understanding
	■ The difference between empathy and sympathy
	■ The process of empathic communication 1. Mindful attention to both verbal and nonverbal messages 2. Empathic simulation of others’ emotion: using one’s own cognitive chain of emotional response 3. Empathic reflection on others’ current emotion and possible causal factors (situation, thoughts, etc.) that might be related to that specific response
	■ Possible obstacles to empathic communication
5^th^ session	■ How to perform empathic communication in the patient-doctor relationship
	■ Empathy in the hospital: facilitator of humanistic connection to patient care
	■ Review, wrap-up, and program evaluation

## Results

### Empathic communication network, constructed from the self-report measures to assess the effects of the EEPMS

This network analysis covered four self-report questionnaires completed by 40 participants who completed the EEPMS (age = 23.5 ± 2.6 (mean ± SD); M/F = 15/25). Their scores on the DASS-21: depression [3.68 ± 4.21 (mean ± SD) before EEPMS vs. 2.65 ± 2.38 after EEPMS], DASS-21: anxiety [2.45 ± 2.99 (before) vs. 2.33 ± 2.53 (after)], DASS-21: stress [7.28 ± 7.80 (before) vs. 5.58 ± 3.92 (after)], EQ-short [18.4 ± 6.70 (before) vs. 19.8 ± 5.52 (after)], JSE-S [75.33 ± 9.15 (before) vs. 76.4 ± 11.69 (after)], and EES [40.58 ± 11.35 (before) vs. 40.6 ± 9.99 (after)] did not show significant changes after the EEPMS (all *p* > 0.05, paired t-test). Based on the criterion of the presence 12 or more cases (=30% of the total participants [*n* = 40]) with responses of ‘absence/no’ or ‘presence/yes’, a total of 24 items (=nodes) were selected from the DASS-21 (two items for depression, one for anxiety, and four for stress), the EQ-short (six items), the JSE-S (one item), and the EES (ten items). These items were used to estimate Ising model-based empathic communication networks (at baseline and after training separately) comprising emotional regulation, empathic understanding for others’ emotion, and emotional expressivity (Table 2). On the other hand, respondents responded to most JSE-S items with ‘accordant’, as shown by their higher total scores at both baseline and at follow-up; therefore, these items were not selected as nodes for the construction of the Ising network (based on *l*_1_-regularized logistic regression). The binarized item-level responses for the 24 selected nodes were used to create the Ising model-based empathic communication network.

**Table 2:** Item labels and distribution of responses for 24 nodes [selected from the DASS-21, EQ-short, JSE-S, and EES for which more than twelve (=30% [n = 40]) cases were detected for ‘absent’ and ‘present’ responses] comprising the empathic communication network.

Measure/subscale	Item	Label	Presence [*n* = 40]	
			Before^a^	After^a^
DASS-21: depression	I find it difficult to work up the initiative to do things.	DASS-21: 5	21	22
	I am unable to become enthusiastic about anything.	DASS-21: 16	16	15
DASS-21: anxiety	I am worried about situations in which I might panic and make a fool of myself.	DASS-21: 9	19	17
DASS-21: stress	I tend to over-react to situations.	DASS-21: 6	27	23
	I find myself getting agitated.	DASS-21: 11	22	23
	I find it difficult to relax.	DASS-21: 12	26	23
	I am intolerant of anything that keeps me from getting on with what I am doing.	DASS-21: 14	22	19
EQ-short: empathy	I can easily tell if someone is masking their true emotion.	EQ-short: 2	21	22
	Other people tell me I am good at understanding how they are feeling and what they are thinking.	EQ-short: 4	16	21
	I can tune into how someone else feels rapidly and intuitively.	EQ-short: 5	20	24
	I am good at predicting how someone will feel.	EQ-short: 7	20	24
	I am good at predicting what someone will do.	EQ-short: 8	16	22
	I can pick up quickly if someone says one thing but means another.	EQ-short: 9	22	25
JSE-S: empathy	Because people are different, it is almost impossible for physicians to see things from their patients’ perspectives.	JSE-S: 6	20	14
EES: emotional expressivity	People think of me as an unemotional person.	EES: 2	18	16
	I don’t express my emotions to other people.	EES: 3	17	15
	I am often considered indifferent by others.	EES: 4	16	14
	Even when I’m experiencing strong feelings, I don’t express them outwardly.	EES: 9	18	21
	Other people aren’t easily able to observe what I’m feeling.	EES: 10	14	16
	I keep my feelings to myself.	EES: 11	18	16
	Even if I am feeling very emotional, I don’t let others see my feelings.	EES: 12	17	17
	I can’t hide the way I am feeling.	EES: 13	22	25
	Other people believe me to be very emotional.	EES: 14	14	16
	I am not very emotionally expressive.	EES: 15	20	15

^a^Number of participants with ‘presence/empathic/emotionally expressive’ reports that were binarized from the original Likert scale-based replies.

### Empathic communication network: hub profile & community membership

**Figure 1:**
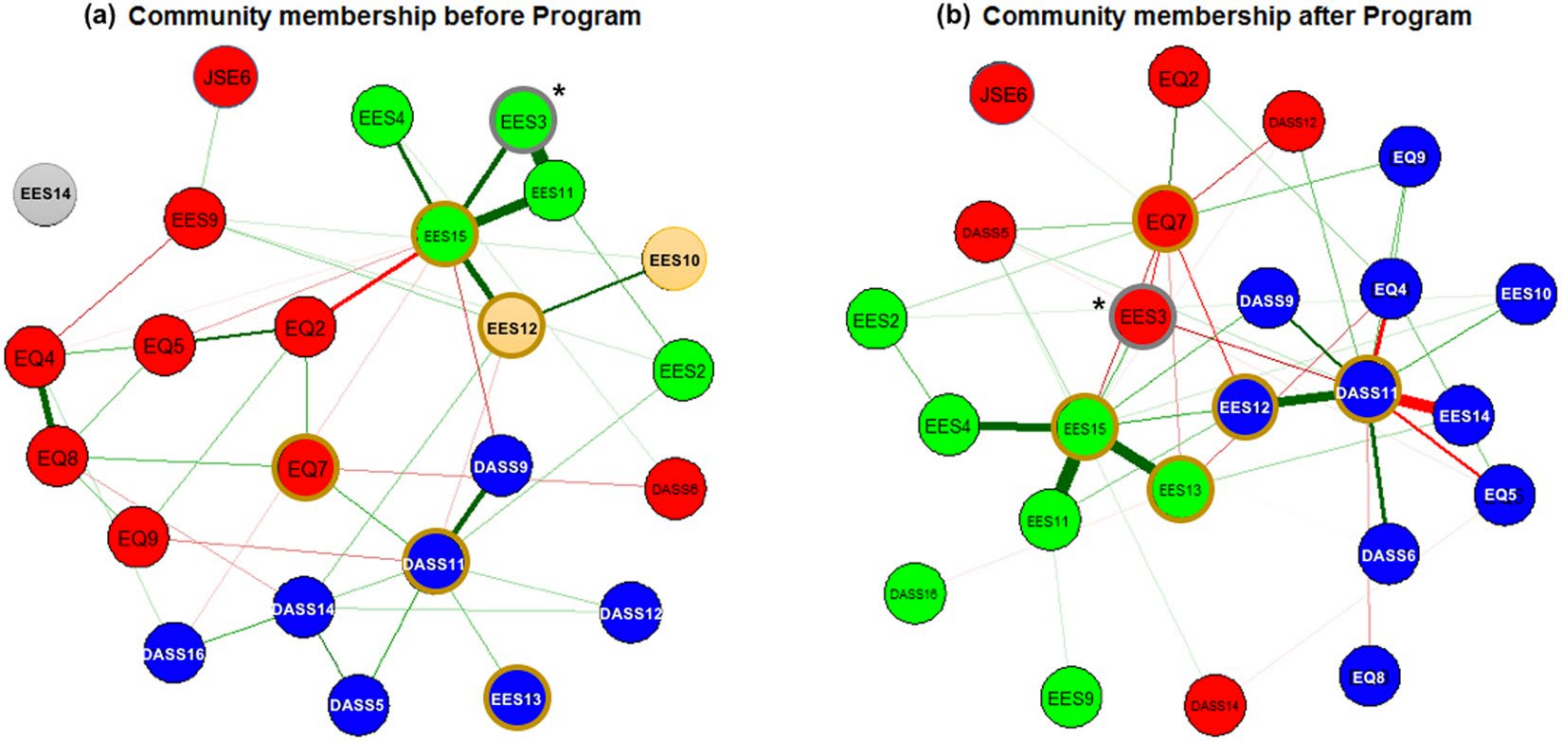
Changed community profiles in the emotion-empathy network (**a**) before and (**b**) after five modules of the Empathy-Enhancement Program for Medical Students. The emotion–empathy networks were estimated using the Ising model; community memberships were detected from the transformed weighted, undirected network using the InfoMap algorithm after the negative connections (red-coloured edges; cf. positive connections tagged with green) were converted into absolute values. Spheres of a given colour identify each distinctive community; among these spheres, a total of five hubs – stress: agitated (DASS-21: 11), empathy: predict feelings (EQ-short: 7), not showing even very intense feelings (EES: 12), cannot hide feelings (EES: 13), and not very emotionally expressive (EES: 15) – are indicated with tan-coloured circles. The node identified by a grey-coloured circle (‘I do not express my emotions to other people’ (EES: 3)) demonstrated a significant change in the clustering coefficient value (*p < 0.015, based on the distribution of values calculated from the graph theory analyses for 5,000 pseudo-networks, produced using random permutations for 80 participant time points into two subgroups). Abbreviations: DASS, Depression Anxiety Stress Scale-21; EES, Emotional Expressivity Scale; EQ, Empathy Quotient-Short Form; JSE, Jefferson Scale of Empathy-S version.

**Figure 2:**
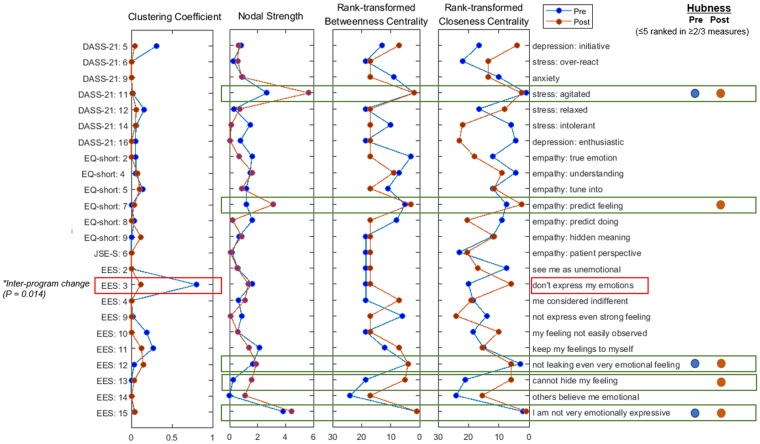
Regional network characteristics of the clustering coefficient, nodal strength, betweenness centrality, and closeness centrality values before (blue-coloured dots) and after (brown-coloured dots) the Empathy-Enhancement Program for Medical Students. The emotion–empathy networks were estimated using the Ising model; the global and regional network characteristics were calculated using the Brain Connectivity Toolbox and Matlab R2016b software after the negative connections were converted into absolute values. As a result, five nodes ranked ≤5 for two of the three centrality measures (node strength, betweenness centrality, and closeness centrality) were selected as hubs (right-hand side of the figure). Moreover, the statistical significance of the changes in the clustering coefficient values was estimated from the distribution of values retrieved from network analyses for 5,000 pseudo-networks (produced by way of random permutations for 80 participant time points into two subgroups) (*p < 0.015). Abbreviations: DASS-21, Depression Anxiety Stress Scales-21; EES, Emotional Expressivity Scale; EQ-short, Empathy Quotient-Short Form; JSE-S, Jefferson Scale of Empathy-S version.

After estimating Ising model-based empathic communication networks (pre vs. post separately; Fig. 1), we retrieved three regional network measures of nodal strength, betweenness centrality and closeness centrality (Fig. 2). Based on the five highest-ranked nodes in two or more of the centrality measures, the top 20%(≈5)-ranked nodes from the 24 initially selected nodes were selected as hubs^31^: (1) DASS-21: 11 [before/after EEPMS: ‘I find myself getting agitated’], (2) EQ-short: 7 [after EEPMS: ‘I am good at predicting how someone will feel’], (3) EES: 12 [before/after EEPMS: ‘Even if I am feeling very emotional, I don’t let others see my feelings’], (4) EES: 13 [after EEPMS: ‘I can’t hide the way I am feeling’], and (5) EES: 15 [before/after EEPMS: ‘I am not very emotionally expressive’].

The InfoMap-based community detection demonstrated changing roles of these five hubs, from intra-domain provincial hubs before EEPMS to inter-domain connecting hubs that integrated and drove the phenomena of stress, empathy, and emotional expression simultaneously after EEPMS (Figs 1 and 2). First, from the local centre of the DASS-21 items, the stress-related hub ‘DASS-21: 11’ changed after the EEPMS to connect items related to stress [‘I over-react to situations’], anxiety [‘I worry about situations in which I might panic and make a fool of myself’], empathy [‘People tell me I am good at understanding how they are feeling and what they are thinking’ and ‘I can tune into how someone else feels rapidly and intuitively; pick up quickly if someone says one thing but means another; am good at predicting what someone will do’], and emotional expressivity [‘Other people are not easily able to observe what I’m feeling; other people believe me to be very emotional’; and ‘Even if I am feeling very emotional, I don’t let others see my feelings’ (another ‘EES: 12’ hub)]. Second, the empathy-related hub ‘EQ-short: 7’ changed from the regional hub of ‘EQ-short’ into a communicator across the domains of depression [‘Difficult to work up the initiative to do things’], stress [‘Difficult to relax; intolerant of anything that keeps me from getting on with what I am doing’], empathy [‘I can easily tell if someone is masking their true emotion’ and ‘It is almost impossible for physicians to see things from their patients’ perspectives’], and emotional expressivity [‘I do not express my emotions to other people’]. Third, the emotional expressivity-related local hub ‘EES: 15’ was also connected to the ‘depression component [unable to become enthusiastic about anything]’ after the EEPMS.

### Changed patterns of the shortest paths between the hub nodes

**Figure 3:**
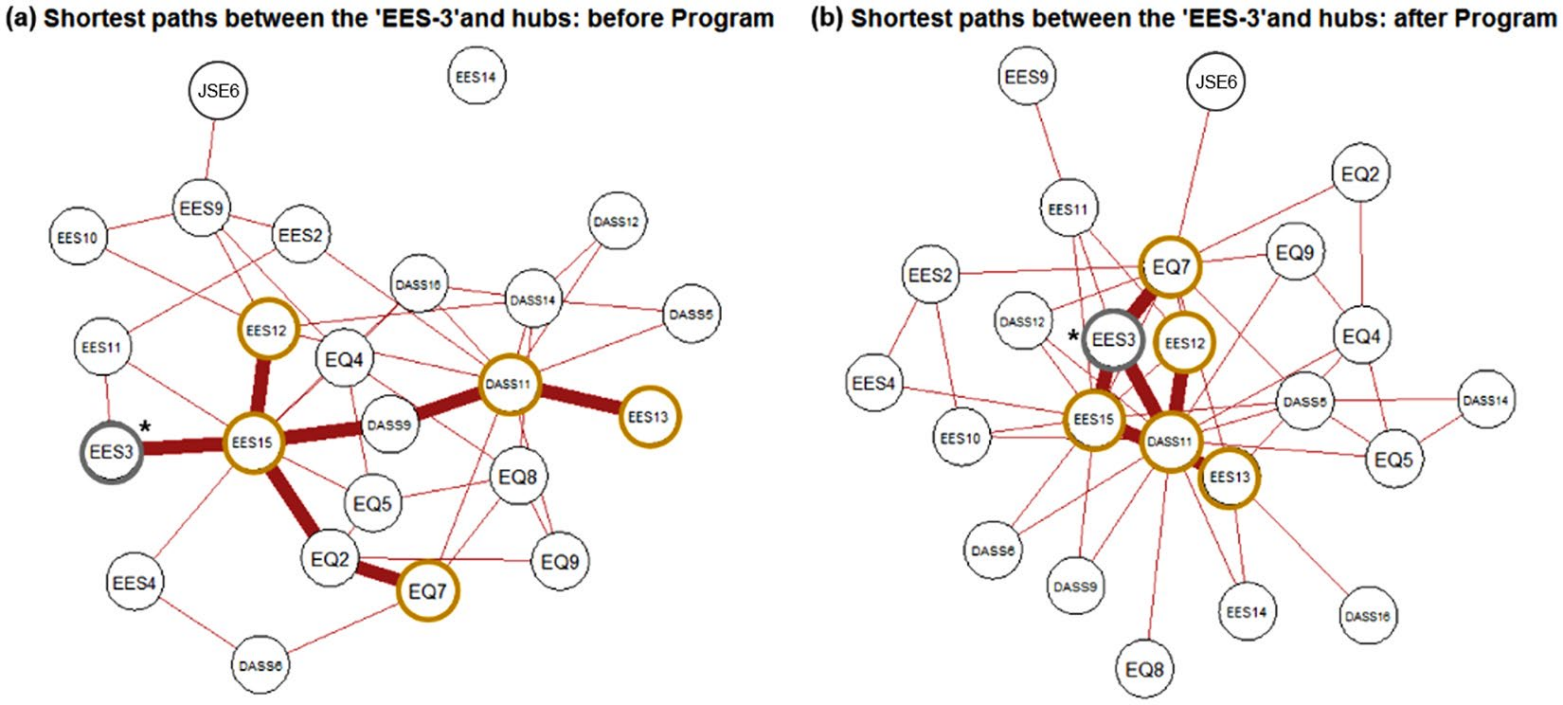
Changed profile of the shortest paths (bold brown edges) connecting the EES: 3 (‘I do not express my emotions to other people’) node with five hub nodes, including DASS-21: 11 (stress: agitated), EQ-short: 7 (empathy: predict feelings), EES: 12 (not showing even very intense feelings), EES: 13 (cannot hide my feelings), and EES: 15 (not very emotionally expressive), in the emotion–empathy network (**a**) before and (**b**) after the Empathy-Enhancement Program for Medical Students. The EES: 3 node demonstrated a significant change in the clustering coefficient value (*p < 0.015, based on the distribution of given values calculated from graph theory analyses of 5,000 pseudo-networks, produced using random permutations for 80 participant time points into two subgroups). Abbreviations: DASS, Depression Anxiety Stress Scales-21; EES, Emotional Expressivity Scale; EQ, Empathy Quotient-Short Form; JSE, Jefferson Scale of Empathy-S version.

The clustering coefficient for the ‘EES: 3’ node [‘I do not express my emotions’]’ in the empathic communication network decreased significantly after EEPMS compared to baseline [*before EEPMS* = 0.802, *after EEPMS* = 0.113; *p* = 0.014 (based on the distribution of given values calculated for 5,000 pseudo-networks, generated using random permutations for 80 participant time points into two subgroups)^41^] after the EEPMS (Fig. 2). The shortest paths connecting the ‘EES: 3’ node to five hub nodes are depicted at two time points, before and after EEPMS, to further explore the changed profile of communication between the ‘EES: 3’ node and important features of the empathic communication network. At baseline (Fig. 3a), the shortest route from ‘EES: 3 [‘I do not express my emotions’]’ to five hubs ran through emotional expressivity-related hub ‘EES: 15 [‘I am not very emotionally expressive’]’. In contrast, after completing the EEPMS (Fig. 3b), the ‘EES: 3’ component revealed a direct connection with stress-related hub ‘DASS-21: 11 [‘I find myself getting agitated’]’, and the emotional recognition-related hub ‘EQ-short: 7 [‘I am good at predicting how someone will feel’]’ as well as the emotional expressivity-related hub ‘EES: 15’. Moreover, the stress-related hub ‘DASS-21: 11’ revealed its role as a connector hub that was directly connected to the three emotional expressivity-related hubs, namely, ‘EES: 12 [‘Even if I am feeling very emotional, I don’t let others see my feelings’]’, ‘EES: 13 [‘I can’t hide the way I am feeling’]’, and ‘EES: 15’, as well as ‘EES: 3’.

## Discussion

This study showed how to quantitatively describe the qualitative item-level effects of the EEPMS. Using a graph theory approach for the Ising model^35^-based empathic communication network, this study uncovered the five most influential components (=hubs) of empathic communication, namely, agitation, self-efficacy for predicting others’ feelings, emotional concealment, active emotional expression, and emotional leakage. Specifically, the key role of the stress component (agitation) uncovered the importance of stress management in preserving the capacity for empathetic communication (Figs 1 and 2). An important facet of this study was the trans-dimensional approach, which encompassed subdomains of empathic communication, including awareness of the importance of empathic patient-doctor communication (JSE-S), self-efficacy with regard to empathic emotional recognition (EQ-short), the tendency to actively express or conceal one’s emotion (EES), and physician-related psychological conditions such as stress, depression, and anxiety (DASS-21). The training effect of EEPMS shown by the attenuated local segregation (=reduced clustering coefficient) and enhanced integration of ‘intentional emotional expressivity’ with these five hubs (=shorter path lengths) suggests that the EEPMS could improve medical students’ recognition of ‘intentional emotional expression’, which is an essential component of physicians’ empathic communication.

### Importance of stress management for preserving empathic communication

The key role of the stress component [DASS-21: 11, agitation] is in line with previous studies that have pointed toward a reciprocal relationship between stress/burnout and empathy among medical professionals^65–67^. Twenty to sixty percent of physicians, including primary and specialized physicians, suffer from job strain and related burnout symptoms^68–73^. Their high workload and inappropriate learning environment are the main sources of distress among medical students, and distress is related to lower empathic capacity during medical training^74^. After repeated encounters with distressing situations, burnout and accompanying cynicism can erode the empathic reciprocal characteristic of patient-physician communication^33,75,76^.

In this study, the post-EEPMS profile of network community membership revealed a closer relationship between the stress-related hub [DASS-21: 11] and diverse components of empathic communication, including anxiety [DASS-21: 9, ‘I am worried about situations in which I might panic and make a fool of myself’], recognizing others’ feelings and thoughts [EQ-short: 4–5, 8–9], and concealing one’s emotion from others [EES: 10, 12, 14]. Accordingly, we infer a possible mechanism of stress-related erosion of empathic communication that is mediated by the loss of calmness (due to agitation), slower and inaccurate reading of others’ thoughts and feelings, a heightened tendency for social anxiety, and concealing one’s emotion. It is not easy for physicians to maintain scientific and medical objectivity while protecting themselves from emotional distress in difficult clinical situations. Indeed, some physicians might try to disconnect from others and develop emotional detachment to defend themselves against distress and burnout^77^. Emotionally detached physicians might depersonalize their patients, become indifferent to patients’ needs, and disregard patients’ feelings^78^.

### The EEPMS effect: cohesive regulation, recognition, and expression of empathic emotions

In this study, a node named EES: 3 [active emotional expression or concealment] showed the most marked effect of EEPMS in terms of the connection with other components in the empathic communication network. The initial membership of the ‘EES: 3’ community was confined only to other EES items (green circles in Fig. 3(a)); among these EES items, the hub ‘EES: 15’ mediated the relationship between ‘EES: 3’ and other parts of the empathic communication network such as social anxiety [DASS-21: 9], hiding self-emotion [EES: 12], and detecting others’ emotional masking [EQ-short: 2] (see bold brown edges around ‘EES: 15’ in Fig. 3(a)). In contrast, the ‘EES: 3’ node was found in the community of emotional regulation-recognition-expression of empathy after the EEPMS (red circles in Fig. 3(b)), connecting all five hubs together. The connection among members of the ‘EES: 3’-related community suggests that medical students who completed the EEPMS would see physicians’ emotional expression [EES: 3] as a necessary component of empathic communication. The emotion expressed by a physician is a product of prerequisite emotional regulation-recognition steps, which include the physicians’ initiative [DASS-21: 5] in taking the patients’ perspective [JSE-S: 6], even in the middle of distress [DASS-21: 12, 14], not only to detect patients’ emotional masking [EQ-short: 2] but also to predict patients’ forthcoming emotional response [EQ-short: 7] in clinical situations. On the other hand, the JSE is designed to measure diverse facets of empathy in relation to patient care, such as perspective taking, standing in the patient’s shoes, and compassionate care, among medical students (S version).

### Limitations

This study has some limitations. First, it examined only the training effects of EEPMS based on the pre- versus post-training measurement, similar to Bryant *et al*.^40^ [in which one group of participants was assessed at two time points after initial exposure to traumatic stimuli] and Seol *et al*.^79^ [in which one group of patients was measured before and after the completion of cognitive-behavioural therapy], and it did not include a comparison between training versus control groups. As a clustering coefficient, the target variable of longitudinal comparison in this study does not follow a normal distribution; therefore, we retrieved *p* values based on the distribution of given values calculated from the graph theory analyses of 5,000 pseudo-networks produced using random permutations for 80 participant time points into two subgroups^41^. In upcoming studies, we will be able to recruit the control groups and will be able to demonstrate the group-by-treatment effect using a nonparametric version of mixed-effect analysis of variance.

Second, even though positive or negative relationships between nodes could have different meanings and could lead to different interpretations, this study regarded these two kinds of relations as the same. However, two centrality measures applied in this study – betweenness centrality and closeness centrality – are capable of being estimated from connection length matrices in which the sign of each edge weight is transformed into the absolute value^62^. We were only able to estimate the degree of influence (regardless of the polarity of relationships) of each component to others comprising the empathic communication network.

Third, due to the paucity of previous studies that applied a network-based approach to examine the training effects for the conceptual integration of multi-dimensional components that comprise empathic communication, we could not estimate the sample size required for this network-based study. Instead, the sample size of this study (*N* = 40) was determined by the number of medical students who voluntarily participated in the five consecutive weekly sessions of extracurricular small-group activities comprising EEPMS from July 2015–July 2017. We hope our study can provide some of the earliest evidence for upcoming network-based studies regarding empathic communication.

Lastly, as respondents responded to most of the items comprising the JSE-S with ‘accordant’, as shown by the higher total scores both at baseline (75.33 ± 9.15) and at follow-up (76.4 ± 11.69), facets of patient care-related empathy reflected in JSE-S such as perspective taking, standing in the patient’s shoes, and compassionate care were sufficiently included in the Ising model-based empathic communication network in this study. Further studies targeting medical personnel with a poorer understanding of the importance of patient care-related empathy might be able to focus more on the role of JSE-S items in empathic communication.

## Conclusions

This study uncovered central components (=hubs) of empathic communication, including agitation, self-efficacy for predicting others’ feelings, emotional concealment, active emotional expression, and emotional leakage. Of note, the key role of the stress component (agitation) in the empathic communication network alerts physicians and medical school organizers of the importance of stress management in preserving the empathic communication capacity. The training effect of EEPMS shown by the reduced local segregation and enhanced integration of ‘intentional emotional expressivity’ with these five hubs suggests that EEPMS could enable medical students to integrate physicians’ emotional expression as an essential component of empathic communication. Further educational efforts by medical schools and training hospitals for effective stress management and empathic communication based on peer discussion, role play, and feedback are warranted.

## Method

### Participants and the EEPMS

The EEPMS was constructed by the research team on the systemization of humanism education in the SNU College of Medicine’^44^, as a revised and condensed version of the Program for Emotional Recognition and Empathic Ability originally developed by Professor Myoung-Sun Roh of SNU. Since 2015, the EEPMS has been regularly administered to medical students by staff (Psychiatrist and Psychologists) of Yeongeon Student Support Centre, SNU College of Medicine (http://yss.snu.ac.kr). The Institutional Review Board of Seoul National University approved the current study. Since this was a minimal-risk study, the written consent of the individual participants was waived by the board.

### Measures: DASS-21

This scale measures negative emotional symptoms. It was originally developed as a 42-item scale by Lovibond and Lovibond^80^, and it was redeveloped by Henry and Crawford^81^ into a 21-item scale that covers three sub-dimensions: stress, depressive symptoms, and anxiety. In this study, the Korean version of the DASS-21, validated by Cha *et al*.^82^, was used. The DASS-21 is scored on a 4-point Likert scale based on the degree of symptoms experienced during the past week (not at all [0] - very much [3]). The responses to each item were binarized into ‘absence (0)’ and ‘presence (1–3)’ to construct the Ising model-based empathic communication network. A paired *t-test* was used to estimate changes in the total DASS-21 score, calculated as the sum of scores on the depression, anxiety, and stress scales of the DASS-21 during the EEPMS (http://www.real-statistics.com/students-t-distribution/paired-sample-t-test/).

### Measures: EQ-short

Based on the Empathy Quotient developed by Baron-Cohen and Wheelwright^54^, Wakabayashi *et al*.^55^ developed a short form of the Empathy Quotient scale, namely, the EQ-short. The EQ-short consists of 22 items reflecting diverse aspects of empathy, including the cognitive, emotional, and social subdomains. This study used the Korean version of the EQ-short validated for nursing students by Yeo^56^, in which only 11 items were selected (numbers 1, 6, 9, 10, 13, 14, 16, 18, 19, 20, and 21) from Wakabayashi *et al*.^55^. A paired *t*-test was used to estimate changes in the EQ-short total scores during the EEPMS. In addition, the degree of participant agreement with each item was scored on a 4-point Likert scale (not at all [0] to very much [3]) and then dichotomized into ‘no empathy (0–1)’ and ‘empathic (2–3)’ for subsequent construction of the Ising model-based empathic communication network.

### Measures: JSE-S

The JSE is a 20-item self-report questionnaire designed to measure diverse facets of empathy in relation to patient care, such as perspective taking, standing in the patient’s shoes, and compassionate care, among medical students (S version) or clinicians (HP version)^57,58^. This study used the Korean version of the JSE-S validated for medical students^59^. A paired *t*-test was used to estimate changes in the JSE-S total score during the EEPMS. Responses for each item were scored using a 7-point Likert scale (very discordant [1] - fully accordant [7]) and were then binarized into ‘no empathy (1–3 for ordinary items and 4–7 for reversed items 1, 3, 6, 7, 8, 11, 12, and 14)’ and ‘empathic (4–7 for ordinary items, and 1–3 for reversed items)’ to further construct the Ising model-based empathic communication network.

### Measures: EES

Kring *et al*.^60^ developed the EES, a self-report measure that addresses several forms of perspective and emotional expressiveness, including expressing emotion (whether positive or negative) outwardly (by way of facial expression, tone of voice, and gestures, among others). The EES is composed of 17 items assessing respondents’ recognition of their own external emotional expression (e.g., ‘Even when I am experiencing strong feelings, I do not express them outwardly’] and others’ judgements of their external emotional expression (e.g., ‘People can “read” my emotions’). In this study, a Korean version of the EES standardized for college students in Korea was used^61^. Responses were initially collected using a Likert scale ranging from ‘never true [1]’ to ‘always true [5]’ and were subsequently binarized into ‘no expression (1–2 for ordinary items and 3–5 for reversed items 2, 3, 4, 7, 9, 10, 11, 12, 15, 16, and 17)’ and ‘emotionally expressive (3–5 for ordinary items and 1–2 for reversed items)’. Changes in the EES total score during the EEPMS were assessed using a paired *t*-test.

### Estimation of the empathic communication network using the Ising model

In the Ising model, every node of a given network exists only in a binary state, and its influence is restricted to direct neighbours; these influences or interactions (=edges) between nodes are estimated using *eLasso*^32^. In this study, based on the criterion of the presence of >12 cases (=30% of total participants [*N* = 40]) for responses of ‘absence/no empathy/no expression’ as well as ‘presence/empathic/emotionally expressive’, a total of 24 items were first selected. Second, from the table of binarized responses per participant for these 24 variables (before and after the program separately), the empathic communication networks were estimated using the R package *IsingFit* (https://cran.r-project.org/web/packages/IsingFit/index.html). After the edges with negative weights were converted into their absolute values, these two weighted-undirected networks (before and after the program separately) subsequently underwent graph theory analyses.

### Graph theory analyses of the empathic communication network: Community detection

Community membership refers to groups of nodes whose shared connections are denser than their connections with non-members. This allows for the determination of whether the network is composed of a single logical structure or a plurality of interacting elements^38^. Among the various community detection algorithms reported, the InfoMap algorithm^83^ is one of the best-performing algorithms available^84–86^. The InfoMap algorithm uses a repetitive network partitioning procedure that closely follows the Louvain method^87^ to detect a hierarchically structured community membership that minimizes the map equation (i.e., the description length of a random walker’s movement in a given network) as an optimal solution^88^. In this study, the optimal community membership of empathic communication networks was detected using a two-level InfoMap algorithm implemented in the *MqpEqation* framework (http://www.mapequation.org). The community membership for empathic communication networks (Fig. 1) was visualized using the R package *qgraph* (http://sachaepskamp.com/qgraph).

### Graph theory analyses of the empathic communication network: Centrality and hubness

To measure the relative importance or influence of specific nodes in a given network, this study applied the notion of centrality. Three centrality measures (Fig. 2), namely, node strength, betweenness centrality, and closeness centrality, were calculated^34,38,89^ using the ‘strengths_und.m’ (for node strength) and ‘betweenness_wei.m’ (for betweenness centrality) functions in the Brain Connectivity Toolbox (https://www.nitrc.org/projects/bct/) and the ‘centrality.m’ (for closeness centrality) function implemented in Matlab R2017a (https://kr.mathworks.com).

### Graph theory analyses of the empathic communication network: Clustering coefficient and changed profiles of shortest paths across program participation

To illustrate the effect of EEPMS on interacting patterns among the important psychological features constituting the empathic communication network, this study focused on nodes that showed a significant change in the clustering coefficient, calculated using the ‘clustering_coef_wu.m’ function in the Brain Connectivity Toolbox, after the EEPMS (*p* < 0.015; based on the distribution of given values calculated from graph theory analyses of 5,000 pseudo-networks, produced using random permutations for 80 participant time points into two subgroups)^41^. Therefore, in this study, the shortest paths between the EES: 3 (‘I do not express my emotions to other people’; *p values for the changes in the clustering coefficient across EEPMS* = 0.014) and five hub nodes in the empathic communication network before and after EEPMS were computed using the ‘distance_wei_floyd.m’ (based on the Floyd-Warshall algorithm) function in the Brain Connectivity Toolbox. Two kinds of shortest paths [before/after EEPMS] connecting the node EES: 3 to hub components (Fig. 3) were visualized using the R package *qgraph* (http://sachaepskamp.com/qgraph). The layout of the empathic communication network was optimized using the Fruchterman-Reingold algorithm^90^.

## Data Availability

The authors will make materials, data and associated protocols promptly available to readers without undue qualifications in material transfer agreements.
